# Bursting out: linking changes in nanotopography and biomechanical properties of biofilm-forming *Escherichia coli* to the T4 lytic cycle

**DOI:** 10.1038/s41522-021-00195-7

**Published:** 2021-03-17

**Authors:** Shiju Abraham, Yair Kaufman, François Perreault, Ry Young, Edo Bar-Zeev

**Affiliations:** 1 Zuckerberg Institute for Water Research, The Blaustein Institutes for Desert Research, Ben-Gurion University of the Negev, Sde Boqer Campus, Midreshet Ben-Gurion, Israel; 2 School of Sustainable Engineering and the Built Environment, Arizona State University, Tempe, AZ USA; 3 Department of Biochemistry and Biophysics, Texas A&M University, College Station, TX USA; 4 Texas A&M AgriLife, College Station, TX USA

**Keywords:** Cellular microbiology, Biofilms

## Abstract

The bacteriophage infection cycle has been extensively studied, yet little is known about the nanostructure and mechanical changes that lead to bacterial lysis. Here, atomic force microscopy was used to study in real time and in situ the impact of the canonical phage T4 on the nanotopography and biomechanics of irreversibly attached, biofilm-forming *E. coli* cells. The results show that in contrast to the lytic cycle in planktonic cells, which ends explosively, anchored cells that are in the process of forming a biofilm undergo a more gradual lysis, developing distinct nanoscale lesions (~300 nm in diameter) within the cell envelope. Furthermore, it is shown that the envelope rigidity and cell elasticity decrease (>50% and >40%, respectively) following T4 infection, a process likely linked to changes in the nanostructure of infected cells. These insights show that the well-established lytic pathway of planktonic cells may be significantly different from that of biofilm-forming cells. Elucidating the lysis paradigm of these cells may advance biofilm removal and phage therapeutics.

## Introduction

Caudovirales, the dsDNA tailed phages that dominate the virosphere, are being developed as alternative therapeutics against multi-drug-resistant bacterial infections^1^. The lysis event that terminates the Caudovirales infection cycle has been studied primarily in model phages of planktonic *E. coli* cultures, especially lambda and T4^2^. The *E. coli* host is a Gram-negative bacterium, comprising an ~45-nm-thick cell envelope that includes a peptidoglycan layer sandwiched between inner and outer membranes^3^. *E. coli* bacteria are ubiquitous in the environment and, in the planktonic state, these cells are usually harmless to humans. However, some *E. coli* strains have acquired the ability to form pathogenic biofilms that cause a broad spectrum of diseases^4,5^. In the initial stages of *E. coli* biofilm formation, attached cells secrete extracellular polymeric substances (EPS) that act as a sticky scaffold for anchoring to the conditioned surface and to each other^5,6^. Once developed, *E. coli* biofilms are notoriously resistant to removal by antibiotics due to the protective barrier provided by the EPS matrix^5^.

An alternative to antibiotic treatment for *E. coli* biofilms is the use of bacteriophage, especially since many coliphages are equipped with virion-mounted enzymes that can degrade elements of the EPS matrix^7,8^. However, details of the phage lytic cycle in biofilm state are lacking, especially in terms of host lysis and virion dissemination. Microscopic real-time imaging has indicated that in the planktonic state, infected *E. coli* cells lyse explosively, usually from a single point on the rod-shaped cellular envelope^9^. Results from biochemical studies, genetics, and fluorescence microscopy have led to a three-step model for the lysis pathway, in which different phage-encoded proteins called holins, endolysins, and spanins sequentially target each of the three layers of the *E. coli* envelope^10^. After the temporally regulated degradation of the peptidoglycan layer, the final step in this lytic cycle is proposed to be localized fusion of the inner and outer membrane. This last step in the lytic pathway leads to the catastrophic explosion of the planktonic *E. coli* cell.

Although the lysis event has been well-studied operationally through the use of video-microscopic as well as molecular and physiological methods^2,11,12^, there is little information on the impact of the lytic pathway on biomechanics and physical structure of the infected cell. During the last decade, several researchers have used atomic force microscopy (AFM) to characterize the infection cycle of several *E. coli* phages. Initially, AFM imaging of infected and dehydrated *E. coli* revealed that by ~30 min after T4-phage infection, the cell envelope has undergone various topographical changes^13^. Later, AFM imaging in more physiologically relevant conditions was done for cells infected with the nonlytic filamentous phage M13, where the progeny are extruded through the intact envelope^14^. Although no change in cell morphology was detected, force vs. indentation measurements have shown that the Young (elastic) modulus of these *E. coli* cells decreased by ~57% after M13 phage infection^14^. For lytic infections, quantitative and kinetic information over the mechanical properties of bacteria is lacking, despite the centrality of this process in phage therapeutics. Moreover, most of the molecular and cellular studies to date have been done with planktonic cells, and there are reports indicating that the phage infection pathway may be significantly different in biofilms^15,16^.

Here, we characterized the nanotopography and analyzed the biomechanical properties of *E. coli* cells that were infected by T4 phages while in the process of forming a biofilm. Using bio-AFM, we acquired nanotopography images of the *E. coli* envelope as well as force vs. indentation curves of the entire cell in real time under physiological conditions. These measurements allowed us to calculate the elastic modulus of *E. coli* cells before, during, and after T4 infection. Our results provide a direct link between changes in the envelope and cell structure to biomechanical properties of *E. coli* cells during the T4 lytic cycle.

## Results

### Changes in nanotopography of biofilm-forming *E. coli* cells during T4-phage infection

**Fig. 1:**
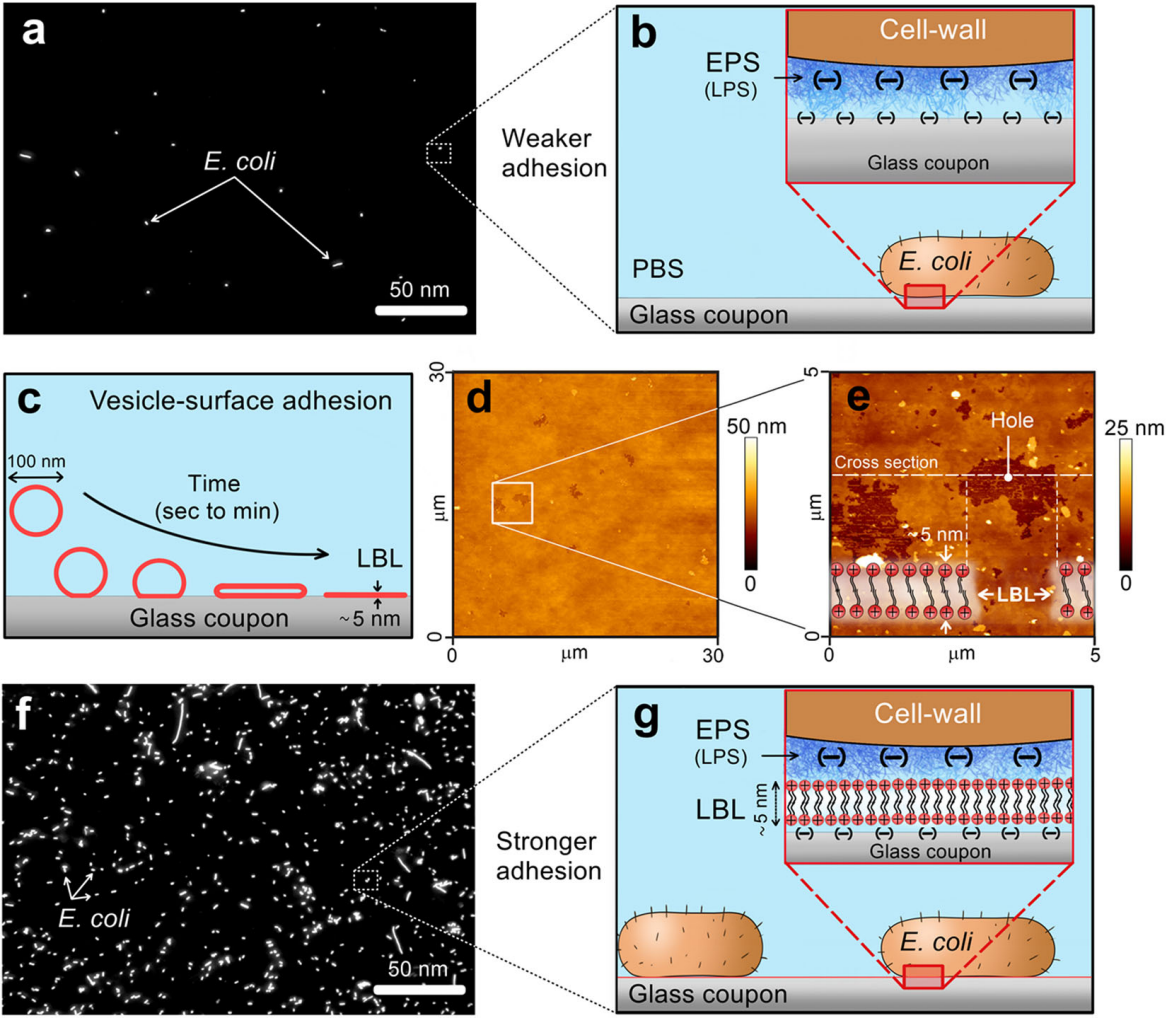
The procedure used for *E. coli* attachment to AFM coupons. Cells were attached to an AFM glass coupon via a positively charged lipid bilayer (LBL), which served as a rigid and adhesive support. **a** Fluorescence micrograph of a sparse *E. coli* cell coverage on a glass coupon without LBL coating. **b** Schematic illustration of the molecular interactions between *E. coli* cells and the glass coupon, pointing on repulsive electrostatic interactions together with weak van der Waals adhesion between the extracellular polysaccharide substances (EPS) and the surface. **c** LBL was prepared on an AFM glass coupon using vesicle fusion techniques, where lipid vesicles adhere to the surface, rupture, and form an LBL coating. **d**, **e** AFM images (in physiological solution) of positively charged LBL on an AFM glass coupon with a corresponding illustration of the LBL coating. Holes in the LBL allowed us to measure the layer thickness. **f** Fluorescence images of *E. coli* cells attached to a glass coupon that was precoated by a positively charged LBL. **g** Schematic illustrations that exhibit the electrostatic adhesion that attaches *E. coli* cells to the positively charged coated surface.

*E. coli* culture was grown on a glass AFM coupon that was precoated with an ~5-nm thin, rigid^17^, and positively charged LBL (Fig. 1). Over a few hours (8 h), *E. coli* cells were irreversibly anchored to the AFM coupon (~1 × 10^6^ cells cm^−2^), with some developed into monolayer clusters, which are early stages in biofilm formation^18^. Irreversible cell attachment to the LBL glass coupon enabled us to capture real-time AFM images and acquire force measurements in a physiological solution. In contrast, cells that came into contact with the glass coupon without the LBL were sparsely attached and dislodged from the surface by the AFM tip, thus hindering image acquisition and force measurements. Control experiments using live–dead staining indicated that attachment to the LBL had no impact on *E. coli* viability (Supplementary Fig. 1). Moreover, additional epifluorescent and AFM images of uninfected *E. coli* over time (*t* = 4 h) indicated that cells were irreversibly attached and formed small (~30 µm^2^) microcolonies (Supplementary Fig. 2).

**Fig. 2:**
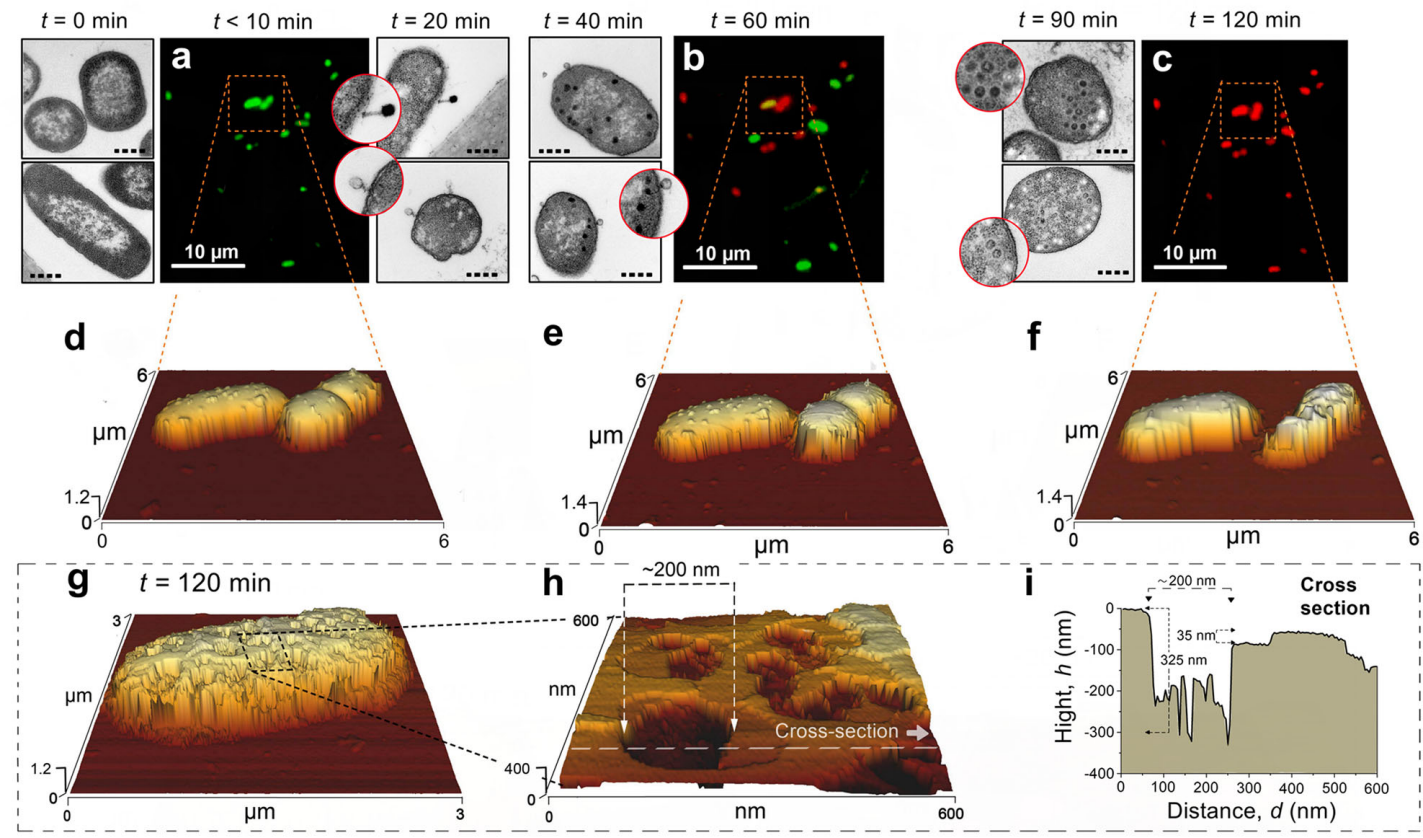
Changes in the physiological properties of *E. coli* cells following phage infection. **a**–**c** In situ fluorescence and **d**–**h** concomitant AFM images following the infection of biofilm-forming *E. coli* cells by T4 phages. **i** Cross-sectional analysis of the corresponding AFM nanotopography image (**h**). Epifluorescence images of “live” (green) and “dead” (red) cells, as well as the corresponding AFM scans, which were captured immediately after T4 infection (*t* < 10 min) as well as 60 min and 120 min following T4 addition. “Live” and “dead” bacteria were identified by staining the cells with SYTO 9 and PI, respectively (see also Supplementary Fig. 1). Complementary microtome slices of *E. coli* cells on a polycarbonate surface covered with LBL were captured by TEM before T4 addition as well as 10, 20, 40, and 90 min after T4 were added. Black circles within the TEM inserts (*t* = 40, 90 min) indicate the presence of T4 virions inside the cells. TEM scale bar was 1 µm (dashed line) for the low magnification and 0.4 µm (circles) for the larger magnifications. Similar images were captured from 12 other cells at 5 individual experiments.

**Fig. 3:**
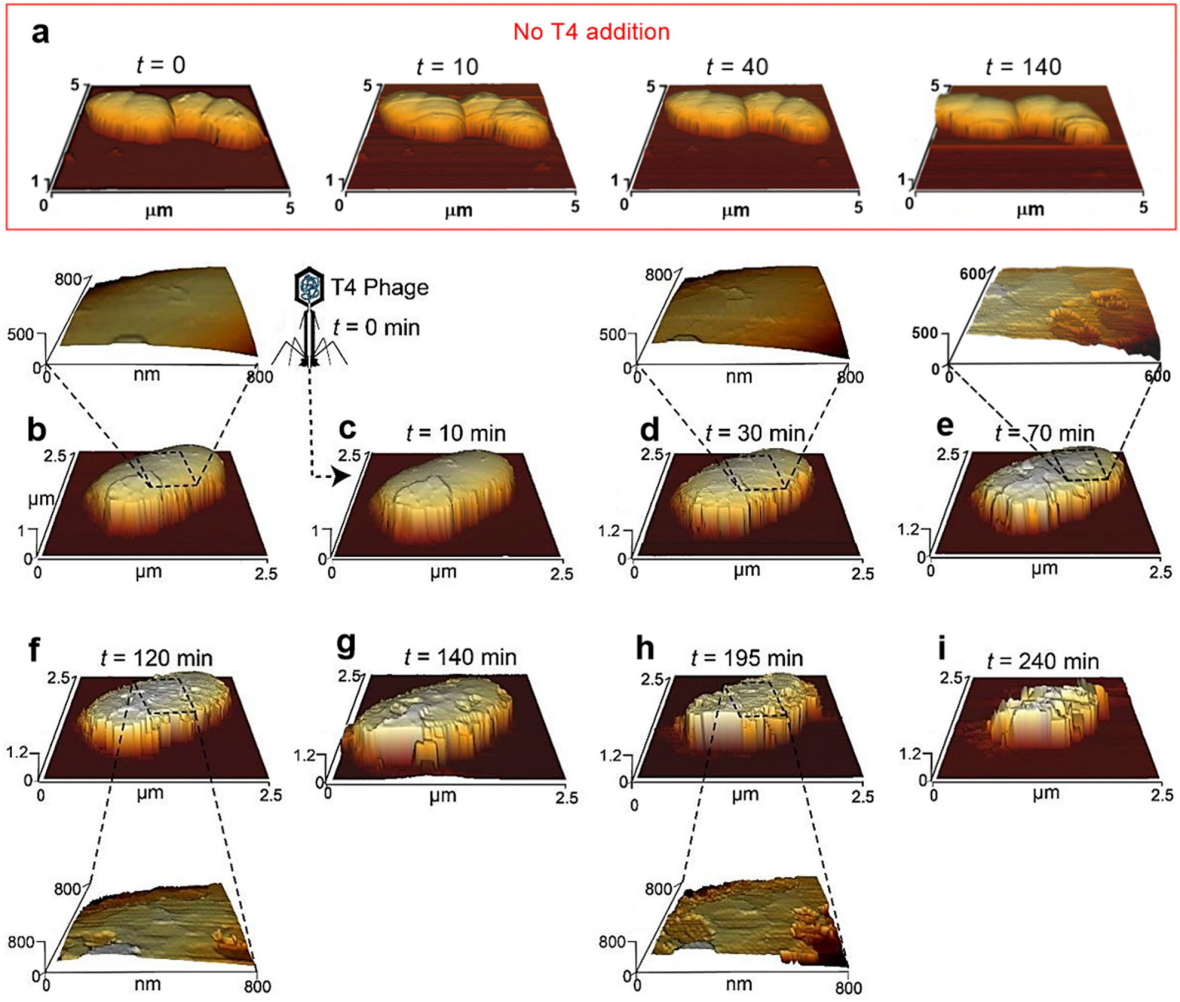
Three-dimensional, time-series images of an *E. coli* cell during T4 phage infection captured in situ by AFM. **a** Images of uninfected *E. coli* cells, namely with no addition of T4. **b** The first image was captured before the addition of T4 phages, defined as *t* = 0. **c**–**i** Time series of AFM images that show the changes in nanotopography of the same cell during the infection cycle under hydrated conditions. Representative sections were enlarged to identify distinct nanostructures at different time points (**b**, **d**, **e**, **f**, **h**). Similar images were captured from 12 other cells at 5 individual experiments.

The topography and viability of irreversibly attached *E. coli* cells before and after T4 infection were studied in situ by epifluorescence microscopy and AFM as well as transmission electron microscopy, TEM (Figs 2, 3). The nanotopography of attached cells was initially captured by AFM prior to T4 addition (defined hereafter as *t* = 0). Phages (2.5 × 10^8^ ml^−1^) were then added together with a Live/Dead staining kit that fluorescently labeled (live, green and dead, red) the attached cells. From this initial stage and up to 10 min after the addition of T4 phages, all the cells exhibit green fluorescence (“live”), indicating that the cell membranes were intact (Fig. 2a). Three-dimensional AFM image analysis of uninfected bacteria indicated that cell size did not change in comparison to their dimensions prior to T4 addition, with an average width of ~1 µm and a length that ranged from 2 µm to 3 µm (Supplementary Fig. 3). We note that phages were not detected on the *E. coli* cells by AFM under these in situ conditions, probably because the AFM tip has physically removed the T4 phages from the surface of the cells during the scan. However, to confirm that T4 phages were able to attach to the *E. coli*, TEM images from parallel subsamples (grown simultaneously under similar conditions) were used as proxy to confirm that successful infection occurred. These images (Fig. 2a, right panels) indicate that 10–20 min after phage addition, one to two phages were attached per cell. Few of these phages were found to include nucleic acids (i.e., black capsid), while others appear contracted and empty of the genetic material (i.e., white capsid) as it was likely injected into the host.

By one hour after the addition of T4 phages, both “live” (green) and “dead” (red) cells were captured, indicating that the cytoplasmic membrane of the latter has been permeabilized (Fig. 2b). In some cells, yellow fluorescence was also detected, presumably reflecting that the membrane has been permeabilized but the PI stain has not yet completely reduced the SYTO 9. The fluorescence intensity of cells that were stained by SYTO 9 increased with time, namely, brighter at *t* = 60 min (50% ± 25%) compared to *t* = 0 (Fig. 2a, b), indicating that phage DNA replication was robust. Complement AFM nanotopography images indicated that *E. coli* cells were mostly intact with no significant change to their size (Fig. 2e). Yet, during these stages (40–90 min), up to 20 virions were detected within 20–60% of the cells by TEM (Fig. 2b, c, left panels).

Two hours after T4 addition, most of the cells were stained with PI (red, Fig. 2c) or completely demolished (Fig. 2f). Concomitantly, AFM images indicated that *E. coli* cells were structurally deformed compared to the same cells at *t* = 60 min (Fig. 2f). Moreover, the nanotopography of infected bacteria at *t* = 120 min shows that cell envelopes were perforated with distinct lesions of 100–300 nm and up to ~350 nm in depth (Fig. 2g–i). Often, the lesions/ruptures had shallow ledges (few tens of nm) with much deeper crevices that may reach a few hundreds of nm (Fig. 2 h, i). It should be noted, according to AFM images of uninfected *E. coli* bacteria, no damage to the cell envelope was caused by the AFM tip during imaging (Fig. 3a).

Dynamic changes in the nanotopography of the cell structure during T4-phage infection were observed in situ by sequential AFM images (Fig. 3). Uninfected cells remained similar with no changes to the nanotopography of the cell envelope or growth over two hours (Fig. 3a). This was also confirmed by Live/Dead imaging, where most of the noninfected cells show minimal changes in their viability over the course of the experiment (Supplementary Fig. 1). In contrast, during the first 10–30 min after the addition of T4 (Fig. 3c, d), envelope topography was distinctly different compared to uninfected cells (Fig. 3a), as if the cell capsule was removed. Yet, no apparent changes to envelope nanotopography were captured (Fig. 3c). However, 70 min after the addition of T4, superficial ruptures (diameter of 105–190 nm and 10–15-nm depth) were visualized in the cell envelope (Fig. 3e). More cavities accumulated over the course of the infection (Fig. 3f–h), ultimately culminating in cells with irregular boundaries and dimensions less than half of the original cell (Fig. 3I).

### Biomechanical changes of *E. coli* cells during T4-phage infection

**Fig. 4:**
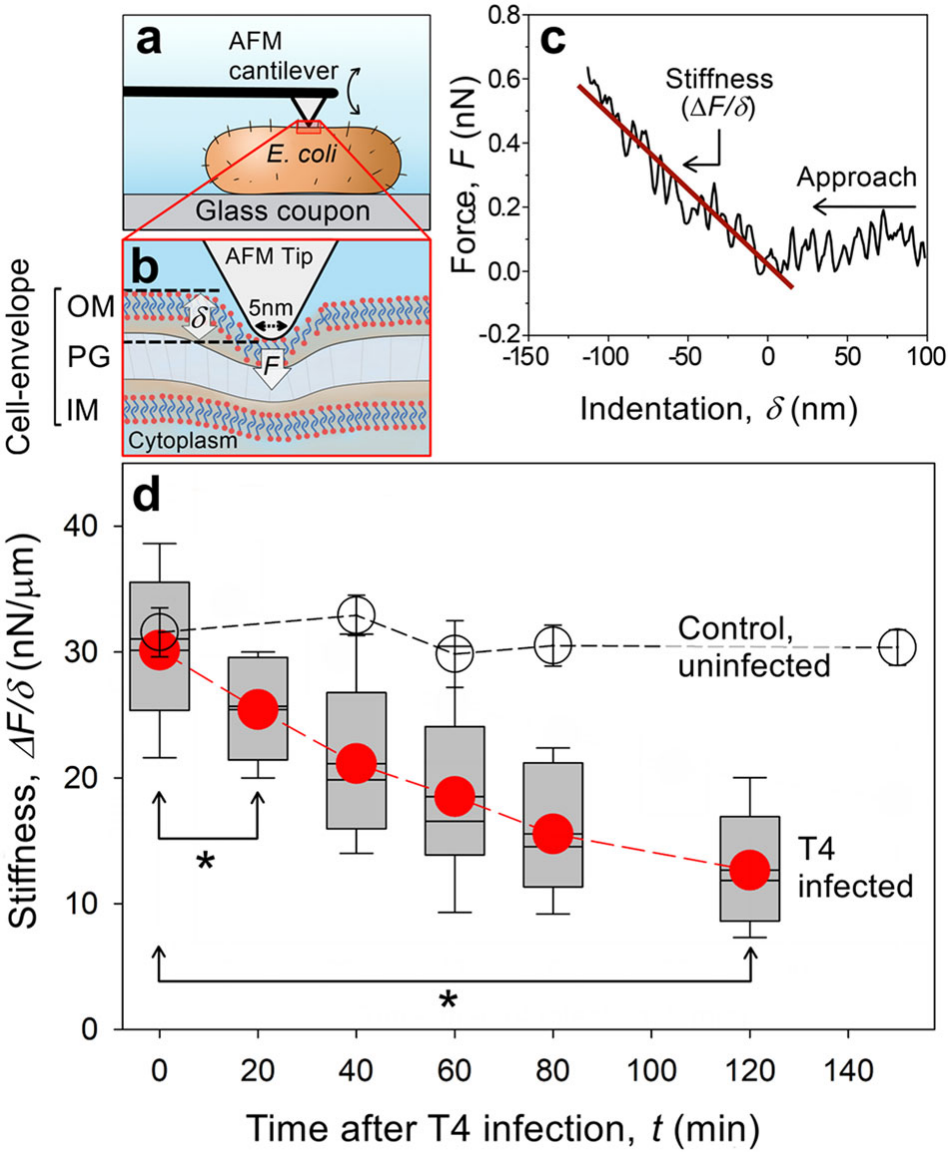
Stiffness measurements of the *E. coli* cell envelope during T4 infection. **a**, **b** Schematic illustration of AFM stiffness measurement using a pyramid AFM tip (not to scale). The force of the nanometer end-tip (*F*) that was applied caused an indentation (*δ*) of the cell envelope, including the outer/inner membrane (OM/IM) and the peptidoglycan layer (PG). **c** A representative AFM force vs. indentation that demonstrates how the stiffness was calculated. Approach velocity was 190 ± 20 µm/s. **d** Stiffness (rigidity) changes with time for different bacteria with (box plot with red circles indicating the average) and without (empty circles) the addition of T4 phages. Average stiffness and the corresponding standard deviation (s.d.) were based on 45,000 force measurements taken from 4 to 5 areas on the surface of each bacterium. Asterisks indicate a statistically significant difference between two time points (*p* < 0.01, *n* = 12 at 3–5 independent experiments).

**Fig. 5:**
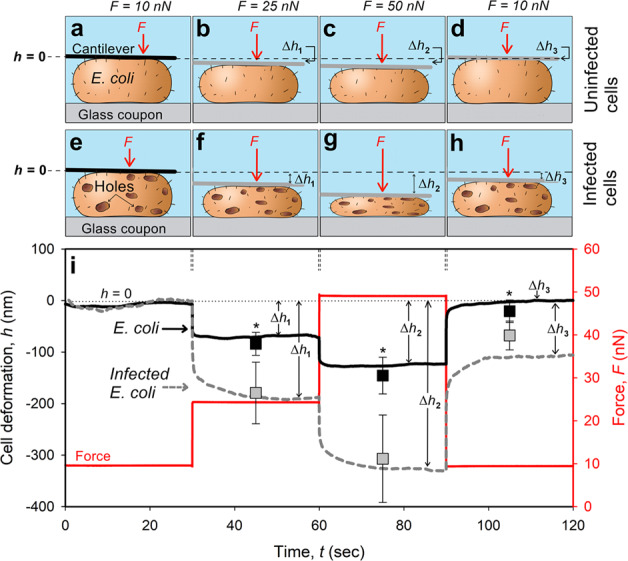
AFM measurements of applied force, *F*, and cell deformation, *h*, vs. time of applied force for infected and uninfected (carried out in separate experiments with no addition of T4 phages) *E. coli* cells. Two hours after the addition of T4 phages, infected *E. coli* were identified and analyzed according to the red fluorescence (i.e., “dead”) of cells with defined structure. **a**–**d** Schematic illustration of the tipless cantilever as it deforms the uninfected and **e**–**h** infected *E. coli* cells at different applied forces. Black full lines **a**, **e** indicate the initial position of the tipless AFM cantilever, while the gray lines **b**–**d**, **f**–**h** mark the position of that cantilever following the application of a specific force. **i** A representative curve where the deformation (indentation), *h*, was set to zero when the AFM cantilever applied *F* = 10 nN. The deformations of uninfected or infected *E. coli* cells (Δ*h*_1_, Δ*h*_2_, and Δ*h*_3_) were measured by applying 25 nN, 50 nN, and 10 nN, respectively. Scatterplot indicates the average and s.d. from 12 different bacteria measured at 3–5 independent experiments. Asterisks indicate a statistically significant difference between uninfected and infected *E. coli* cells (*p* < 0.01, *n* = 12).

Changes in the biomechanics of the cell envelope (Fig. 4) and the entire bacterium (Fig. 5) following T4 infection were measured in situ by AFM in a biocell. The stiffness of the cell envelope was calculated as *ΔF/δ*, where *F* is the force that was applied on the cell and *δ* is the indentation of the AFM tip into the cell. Measurements were averaged from a total of ~45,000 data points taken at 4–5 different locations (250–500 nm^2^) on the surface of each bacterium (*n* = 12) during infection (Fig. 4a, b). These force measurements were done using a pyramid AFM tip with a radius of curvature of ~5 nm, and the approach speed was 190 ± 20 µm/s. A significant reduction in cell envelope stiffness (16%, *p* < 0.02) was already detectable 10–20 min after the addition of T4 phages and continued to decrease monotonically, reaching ~58% reduction by 2 h (Fig. 4d). In contrast, no change in cell envelope stiffness was detected over a similar time frame for uninfected bacteria (Fig. 4d).

In addition to cell envelope stiffness, the elasticity (*E*) of a whole cell was calculated based on a strain response (deformation) of a single bacterium to a sudden change in force. These measurements were done in situ using a tipless AFM cantilever, and the equation that was used to calculate *E* is specified in the “Methods” section (Fig. 5, Supplementary Figs 4, 5). Infected bacteria were identified 2 h after the addition of T4 according to the red fluorescence of the cells (i.e., “dead” but intact bacteria). Compatible force profiles were also measured from uninfected (no addition of T4) bacteria two hours after placing the attached *E. coli* in the AFM biocell.

Initial contact between the tipless cantilever and the bacteria was achieved by applying a constant force of 10 nN for 30 s (Fig. 5i). During that time, the deformation of infected and uninfected bacteria was defined as *h* = 0 nm (Fig. 5a, e). A sudden (<1 ms) change in force was then applied (from 10 nN to 25 nN), and *F* maintained constant for 30 s. For uninfected bacteria, this resulted in an initial cell deformation, characterized by a stepwise decrease in cell height, of Δ*h*_1_ = 84 ± 22 nm (Fig. 5b, i). Under the same applied change in force, infected bacteria deformed twice as much (Δ*h*_1_, 179 ± 60 nm). Note, infected bacteria also deformed, with an initial immediate deformation followed by a slow (15–20 s) and gradual decay (Fig. 5f, i), which is an indication of a viscous component that was not observed in uninfected bacteria. Concomitantly, the Young’s modulus (elasticity) of the infected cells was significantly (*p* < 0.01, *n* = 15) lower (46%) than uninfected bacteria (33 ± 13 kPa and 61 ± 32 kPa, respectively). Increasing the applied force to 50 nN (additional step of 25 nN) resulted in a similar trend (Fig. 5c, g), namely, greater (105%) deformation of infected cells was measured compared to the uninfected bacteria (Δ*h*_2_, 307 ± 85 nm and 146 ± 36 nm, respectively). Similarly, the calculated *E* of infected cells was also significantly (*P* < 0.01, *n* = 15) lower (41%) than uninfected bacteria (72 ± 20 kPa and 122 ± 54 kPa, respectively).

Retracting the cantilever to the initial force of contact (10 nN) caused uninfected bacteria to stretch back to their initial thickness (Δ*h*_3_, 21 ± 22 nm, Fig. 5d). In contrast, infected bacteria (with a defined structure, Fig. 5h) remained significantly thinner after relaxing the applied force (Δ*h*_3_, 68 ± 28 nm). Moreover, elasticity following the recovery segment (Fig. 5i, 120 s > *t* > 90 s) remained much lower (48%) for infected cells than uninfected bacteria (56 ± 18 kPa and 107 ± 68 kPa, respectively).

## Discussion

Studies of bacterial cell topography following phage infection have typically been carried at a nanoscale resolution by electron-based microscopy methods that are not suited to real-time imaging^19–22^. During the last decade, cell topography after phage infection has been imaged by AFM-based techniques under dehydrated^13,23^ and hydrated (in situ) conditions^14,24,25^. In addition to nanoscale imaging, AFM has been used to measure changes in biomechanical properties of phage-infected bacteria^14,24^. Still, a major challenge of using in situ AFM to study bacterial samples is attaching the cells to the surface^26–28^. One option is using cross-linking approaches (e.g., EDS-NHS and polydopamine) that immobilize the cells to the surface^29,30^. However, these approaches might affect the physiological characteristics of biological samples. A common approach for biological samples is using a positively charged poly-L-lysine “bio-glue” to attach negatively charged cells with the surface. Unfortunately, it is challenging to control the thickness of the poly-L-lysine layer, which can be several µm thick, thus acting as a soft (cushion-like) support that affects the elasticity measurements of the cells.

Here the cushion effect of poly-L-lysine was avoided by using a rigid (vertical elasticity of ~10 MPa)^31^, positively charged LBL to act as a nanothin glue (Fig. 1). The LBL adheres to *E. coli* cells via electrostatic interactions between the negatively charged lipopolysaccharides^32^ and the positively charged LBL^33^. We note that during imaging and elasticity measurements, the AFM tip exerts vertical and lateral forces on the cells. These forces can move and/or dislodge the cells from the surface, thus impairing the AFM measurements. However, the attachment of *E. coli* cells to the LBL layer under physiological conditions was robust enough to minimize cell dissociation from the surface (Fig. 1), without impacting cell viability (Figs 2a, 3a, S1A, B).

The earliest infection-specific changes (*t* < 10 min) was the increase in SYTO 9 fluorescence, presumably reflecting the accumulation of newly synthesized phage DNA. This is consistent with previous studies showing that, although host DNA is degraded to nucleotides during the early stage of infection, viral DNA biosynthesis is much greater, such that total DNA mass accumulates to levels ~10-fold higher than preinfection^34^. Interestingly, a concurrent reduction in cell envelope stiffness (i.e., outer and inner membranes as well as the peptidoglycan layer) was also measured (Fig. 4). This result was unexpected, since the generally accepted view is that the envelope of the infected cell is not challenged by the multistep lysis program until just before virion release^2^. However, the infection event itself does result in damage to the host envelope. At the infection site, the outer membrane is penetrated by the T4 puncturing device composed of a trimer of gp*5* attached at the end of the tail tube^35–37^. These three gp*5* lysozyme domains are released and catalyze local degradation of the peptidoglycan layer, allowing the tail tube to approach the outer surface of the cytoplasmic membrane^35,37^. Indeed, at high multiplicities of infection (MOI), this can lead to the phenomenon of “lysis from without”^37,38^. However, we surmise that lysis from without did not occur under these conditions as only few phages were found attached to the *E. coli* cells (Fig. 1a, *t* = 20). Nonetheless, it was evident that the infection has progressed even at these low MOI as virions assembled within the cells over time (Fig. 1b, *t* = 40 and c, *t* = 90). It is likely that the few liberated lysozyme domains could do significant damage and may also cause induction of cell wall repair systems. According to the infection paradigm known for planktonic cells, three gp*5* lysozyme domains are liberated in the periplasm per infecting phage; thus, over time, critical damage could be done to the peptidoglycan layer just from these molecules alone. Moreover, the penetration of the tail tube through the outer membrane may induce host envelope maintenance systems that could temporarily alter the physical properties of the cell. In addition, throughout the morphogenesis phase, the spanin complexes connecting the inner and outer membrane accumulate in the envelope^11^. Although the proposed membrane-fusion activity of the spanin complexes is blocked until the peptidoglycan layer is degraded^12^, nevertheless, the spanins comprise a direct protein-mediated link between the membranes. These links could affect the stiffness and elasticity of the host cell envelope. Finally, about half of T4 genes have unknown functions and could be participating in these early effects on the envelope properties^34^. For example, phage lambda has three “moron” loci that modify the envelope early in infection^39–41^. The powerful genetics available for experiments with T4 combined with Bio-AFM imaging should enable us to further examine the relationship between the expression and function of these genes in the early stages of infection.

One to two hours after the addition of T4, structural damages to the cell envelope were detectable by the penetration of PI (red fluorescence) into the cells (*t* = 60–120 min, Fig. 2, S1D). Taken together with the progression of the observed cell population from “live” to “dead” staining over ~60 min, this adds confidence that a robust phage infection was occurring in the infected population. At that time point, numerous nanoscale ruptures (100–300 nm in diameter) on the outer cell envelope (15–40 nm in depth) and through the cell envelope (<300-nm deep) were captured by AFM (Fig. 2h, i). These changes in cell topography are clearly the result of T4 infection as no such changes were detected in uninfected cells (Fig. 2a). Previously, comparable lesions (~350 nm) were found in the inner membrane of planktonic *E. coli* cells after triggering of the T4 holin^19,42^. These inner-membrane holes were found to be formed by the T4 holin (gp*t*) at a programmed time, allowing the T4 endolysin to attack the peptidoglycan layer and thus determine the timing of *E. coli* lysis. In turn, the destruction of the peptidoglycan layer liberates the spanin complexes. According to the current view, this allows them to diffuse laterally, aggregate, and disrupt the structure of the outer membrane by fusion with the inner membrane^2^. This pathway results in explosive, localized lysis in planktonic cells, with most “blow out” events localized to the cell poles in the case of phage lambda^11^. The simplest interpretation of the results of the nanotopographical studies is that in a biofilm state, the outer membrane disruptions occur at random locales around the cell, possibly reflecting the underlying holin lesions in the inner membrane. Whether or not these findings confer an advantage for more effective dispersal of phage progeny from cells in mature biofilms remains to be seen.

Force measurements, complementary to the nanotopographical analysis, indicated dramatic differences in cell elasticity between infected and uninfected bacteria (Fig. 5). Based on numerical fitting of the data to the classical models, such as Kelvin–Voigt, calculation of the host elasticity and viscosity yielded large errors for the viscous component(s) due to low sensitivity of the fitting method to changes in viscosity (Fig. 5i). Therefore, we considered only the elasticity of the *E. coli* cell. It should be noted, however, that in the case of viscoelastic materials (not plastic), viscosity only affects the time at which the strain reaches a steady state, while elasticity determines the strain at equilibrium. Therefore, to calculate the elasticity, *E*, we considered only the initial and final strains (30 s after the sudden change in force). Nonetheless, slower deformation of infected cells under constant forces of 25 nN or 50 nN (Fig. 5i) indicated that infected cells exhibited viscoelastic or even plastic behavior (i.e., cells deformation did not recover after relaxing the stress). On the other hand, uninfected bacteria exhibited pure elastic properties. We propose that the viscoelastic and plastic characteristics of infected cells are due to the production and assembly of new viral DNA during this infection phase^43,44^. We surmise that it is likely that these infected cells remained partly deformed following the gradual loss of cytoplasm via lesions in the cell envelope. Yet, these infected bacteria did not explode during virion release, as the cells were not completely deformed, but instead partly flexed back after relaxing the applied force.

Taking together the results of this study, we suggest that the lysis paradigm of T4, which was commonly studied in cultures of planktonic cells, is markedly different for *E. coli* cells that are in the process of forming a biofilm. Planktonic *E. coli* cells lyse in an explosion that likely disperses the progeny virions. In contrast, irreversibly attached *E. coli* cells, which are forming a biofilm, undergo a gradual (minutes to hours) lysis pathway. More importantly, during the lytic cycle of biofilm-forming cells, the envelope becomes perforated and softer. Specifically, the elasticity of the cells decreases over time, and the cells exhibit more viscous and plastic (unrecovered deformation) behavior than uninfected cells.

The explosive lysis mechanism may provide an ecological advantage for planktonic *E. coli* bacteria, if the dispersal of the progeny reduces the likelihood of readsorption to the envelope debris of the dead cell. However, a more gradual release may minimize the risk of lysis from without events of biofilm-forming *E. coli* as virions are released in close proximity to other attached cells and diffusion into the bulk is limited. Nonetheless, the clear differences in the lytic cycle of biofilm-forming *E. coli*, including changes in the structural and biochemical properties of the cells, identify new knowledge gaps, related to (i) the impact of capsule depolymerase expression in phage infection of multilayered biofilms, (ii) the role of endolysins, holins, and spanins in the lysis of bacterial biofilms, and (iii) the effect of phage infection on the mechanical properties of a biofilm structure. Moreover, elucidating the lysis phenomenon in bacterial biofilms may provide alternative means to enhance the efficiency of biofilm removal and phage therapeutic.

## Methods

### Preparation of a lipid bilayer (LBL) surface to immobilize bacterial cells

AFM glass coupons (25 mm in diameter, Menzel Gläser, MENZCB00250RAC) were thoroughly cleaned with acetone and ethanol followed by Milli-Q water. The AFM coupons were then dried with N_2_ gas and UV-cleaned for 10 min prior to the vesicle fusion on the glass surface. Zwitterionic 1,2-dimyristoyl-sn-glycero-3-phosphocholine (DMPC), and positively charged 1,2-dimyristoyl-3- trimethylammonium-propane (DMTAP) lipids (Avanti Polar Lipids, Inc., Alabama, USA) were used for the LBL coating. NaCl solution (1 ml, 150 mM) was added to the dried lipid combination (DMPC + DMTAP), to reach a total concentration of 0.5 mM lipids, thus resulting in a positive surface charge^33^. Ultrasonication for 2 min (60 Hz, Witeg-Germany), followed by gradual heating (55 °C) for 20 min, was performed to achieve a well-dispersed vesicle solution. The AFM glass coupon was uniformly covered by the vesicle solution (350 µl) and incubated at room temperature for 10 min. During that time, vesicles were fused with the surface, leading to a self-assembled LBL. The residual vesicles were removed by washing with 150 mM NaCl solution.

### Bacteria culture and AFM biocell setup

*E. coli* B strain (ATCC 11303) was cultivated to the exponential phase (OD_600_ of 0.6–0.8) in Luria-Bertani broth (BD244620, Becton, Dickinson and Company, United States) at 37 °C. *E. coli* cells were irreversibly attached by placing a subsample (~600 µL) onto an LBL-precoated AFM glass coupon (Fig. 1). Inoculated AFM coupons were kept in a humid chamber (to minimize cell dehydration) at 37 °C for ~8 h. At the end of the incubation, any loosely bounded bacteria were washed with sterile phosphate buffer saline, PBS (P4417, Sigma, US). Immobilized bacteria were stained and localized by SYTO 9 (final concentration of 0.01 mM, Invitrogen, ThermoFisher Scientific, United States), a fluorescent fluorophore (Ex 485 nm, Em 498 nm), for 20 min in the dark. Excess stain was gently removed by replacing the PBS media three times. The glass coupon with immobilized and stained bacteria was assembled into an AFM biocell according to the manufacturer’s instructions (JPK Instruments, Germany) and filled with sterile PBS (1 ml) (Supplementary Fig. 4). Bacteria were initially localized by an inverted epifluorescence microscope (Zeiss Axio Observer.Z1, Germany) equipped with a green filter (Ex. 470 ± 20 nm and Em. 525 ± 25 nm) and integrated with the AFM. Images of attached bacteria were captured and analyzed in situ and in real time by an AFM prior to the addition of the phages (i.e., *t* = 0) and up to 4 h at different intervals (JPK Instruments AG, Berlin, Germany). Biomechanical properties of infected and uninfected cells were captured ~2 h after T4 addition. Detailed information on AFM imaging and measurements is provided below.

### Propagation of T4 phages and proceeding of the infection experiments

T4 phages (ATCC, 11303-B4) were thawed and propagated according to Adams et al.^45^. Briefly, *E. coli* (1 ml, log phase) was mixed with 50 µL of T4 phage and 5 ml of worm soft agar (30 mg/ml of tryptic soy broth (TSB) + 0.5% agar), poured onto TSA plates, and incubated at 37 °C overnight. Sterile PBS (10 ml) was added into the inoculated LB plate for 1 h at room temperature. PBS and phages (~8 ml) were collected and centrifuged (8000 × *g*) for 15 min. The concentrated phages were filtered through a 0.45-µm filter (SLHVM33RS, Millex-HV Syringe Filter Unit, Merck, Germany) and stored at 4 °C.

Phage concentrations in the stock culture were quantified by counting plaque-forming units (PFU). Briefly, log-phase *E. coli* (1 ml) was mixed with a serial dilution of T4 phages in 5 ml of warm 0.5% TSB agar. The mixed media was poured onto a solid TSB (1%) plate and incubated at 37 °C overnight. Plaques were counted after 12 h. Negative controls (i.e., no addition of T4) were done for all PFU tests.

Following the initial localization of *E. coli* (by SYTO 9 only), T4 phages were added (2.5 × 10^8^ phages ml^−1^) to the AFM biocell (Supplementary Fig. 6) together with SYTO 9 (Ex 485 nm, Em 498 nm) and PI (Ex. 535 nm and Em. 617 nm) (BacLight Bacterial Viability and Counting Kit, Invitrogen, ThermoFisher Scientific, United States) at concentration of 0.06 mM. Live/Dead images of infected cells were first captured by epifluorescent microscopy (~10 min after adding the T4 phages) followed by nanoscale images and the corresponding force measurements of their cell envelope (*t* = 10 min). Similar approach was taken along the rest of the experiment at varying intervals.

### AFM imaging and force vs. distance vs. time measurements

AFM images were acquired using JPKSPM (JPK Instruments AG, Berlin, Germany) with an SNL-10 probe (Bruker, Camarillo, CA) and a spring constant of 0.35 N/m in quantitative imaging (QI) mode at room temperature (25 °C). The AFM force vs. distance vs. time curves of entire cells were measured using a tipless cantilever (MIKROMASCH, Sofia, Bulgaria). The spring constant of the cantilever used here is 0.4 N/m. The deflection vs. displacement vs. time data were converted into force vs. relative distance vs. time graphs using the JPKSPM-data processing software.

Nanoscale images of random cells were captured by the AFM, while successful infection was confirmed in real time via Live/Dead staining. Fluorescent Live/Dead images of the samples on the substrates were captured by an epifluorescent microscope (Axio Zoom. V16, Zeiss, Germany) which is coupled with the AFM. Differently, infected cells were specifically chosen according to Live/Dead staining (appeared red) two hours since the addition of the T4 phages to determine the impact of T4 infection on the biomechanics of the cells.

### Calculation of the cell elasticity using a tipless cantilever

The elasticity (*E*) was calculated by

1 $$1 - \frac{h}{{h_0}} = 1 - \frac{F}{{A \cdot E}},$$

where *h*_0_ is the cell height (thickness) as measured by AFM imaging and *h* is the indentation depth of the tipless cantilever into the cell (see Supplementary Figs 4, 5). *F* is the force that was applied on the cell, as measured by the AFM. *A* is the contact area between the tipless cantilever and the cell. Note, *A* changes with *h* according to Eq. S2. Additional details on the elasticity calculations are provided in Section 4.3 in the Supplementary Information.

### Transmission electron microscopy (TEM)

Similarly to the above procedures, *E. coli* B cells (600 µl) were grown for 8 h on 25-mm, 0.2-µm Supor® PES membrane disc filters (Pall Corporation, USA) covered with LBL (as described above apart from acetone cleaning). We surmised that it is unlikely that the difference between the mechanical properties of the covered glass AFM coupon to the covered disk filter used for TEM had critical impacts over the infection. TEM samples were then placed in a petri dish and incubated in a humid chamber at 37 °C. Loosely attached cells were removed from the membrane by rigors washing with a 10-ml syringe field with sterile PBS. Immobilized *E. coli* cells were inoculated with T4 (final concentration of 5 × 10^7^ phages ml^−1^). At *t* = 0 before phage infection and at 20-min intervals after infection, a filter was fixed with glutaraldehyde solution (2.5%, G7651, Sigma-Aldrich, United States) in a 1.5-ml eppendorf overnight at 4 °C. Fixed samples were stained with osmium tetroxide and sequentially dehydrated according to Bogler and Bar-Zeev 2018^46^. Dried samples were embedded in an epoxy resin (Embed 812, Electron Microscopy Sciences, Hatfield, PA), cut into 70-nm slices, and imaged with a Philips CM12 TEM equipped with a Gatan 791 CCD camera.

### Statistical analysis

Statistical analyses were performed using Microsoft Excel and the data were plotted using Origin 2018 or by SigmaPlot. Standard deviation, averaging, and T-test were conducted by this excel sheet. Independent bacterial samples were measured to determine cell deformation (*n* = 12), cell stiffness (*n* = 12), and elasticity (*n* = 15). Further, confidence level of *p* < 0.01 was required for the *T*-test analyses throughout the paper.

### Reporting summary

Further information on experimental design is available in the Nature Research Reporting Summary linked to this paper.

## Supplementary information

Supplementary Information

Reporting Summary

## Data Availability

All data measured and analyzed during this study are included in the paper.
