# Supplementary material for: Bursting out: linking changes in nanotopography and biomechanical properties of biofilm-forming Escherichia coli to the T4 lytic cycle
Source: NPJ Biofilms Microbiomes. 2021 Mar 17;7:26. doi: 10.1038/s41522-021-00195-7 (PMC7969764; doi:10.1038/s41522-021-00195-7)
Supplement: Supplementary file 1 — Supplementary Information [file 41522_2021_195_MOESM1_ESM.pdf]

Supporting Information

Bursting out: linking changes in nano-topography and biomechanical properties of biofilm-forming Escherichia coli to T4 lytic cycle

Biofilms and Microbiomes

Shiju Abraham[1], Yair Kaufman[1*], François Perreault[4], Ry Young[2,3], and Edo Bar-Zeev[1*]

[1]Zuckerberg Institute for Water Research, The Blaustein Institutes for Desert Research, Ben-Gurion University of the Negev, Sde Boqer Campus, Midreshet Ben-Gurion, 8499000, Israel.

[2]Department of Biochemistry and Biophysics, Texas A&M University, College Station, TX, 77843, USA

[3]Center for Phage Technology, Department of Biochemistry and Biophysics, Texas A&M University, College Station, TX, 77843, USA

[4]School of Sustainable Engineering and the Built Environment, Arizona State University, Tempe, AZ 85287-3005, United States

Supplementary Results and Discussion

Control experiments: Our results indicate that the green florescence ("live") of attached *E. coli* cells to the positively charged lipid bilayer (LBL) was mostly unchanged during 120 min (Fig. S1 A, B panels). However, few bacteria were found to become red ("dead") over time, indicating that some cells were physiologically impaired. Therefore, we concluded via Live/Dead assays that most of the non-infected cells were not affected by the LBL over the course of these 2 hours. In contrast, *E. coli* cells showed an increase in green florescence following 10 to 60 min after the addition of T4 phages (Fig S1 C), which was likely due to the accumulation of intracellular T4. In addition, many cells became red ("dead") following 60 to 120 min after T4 addition, indicating cell wall perforation (Fig S1 D).

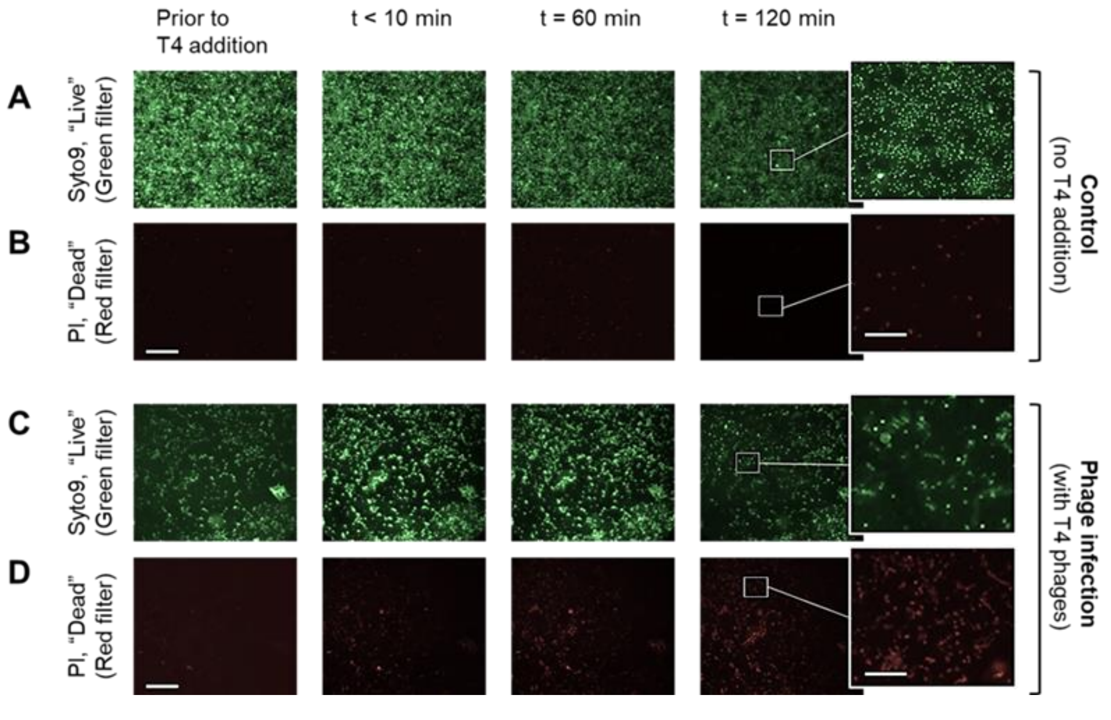

Supplementary Figure 1. Physiological changes of uninfected and infected E. coli cells over time. Live (green) and dead (red) staining of *E. coli* cells that were attached to the positively

charged LBL and captured *in-situ* by epifluorescence microscopy. (A and B) Control experiments were carried without the addition of T4 phages for ~120 min in the AFM bio-cell. (C and D) Concomitant experiments included incubation of attached cells with T4 phages for 120 min. Scale bar of the main plots is 50 μm and of the inserts is 20 μm.

Over four hours since the begging of the experiment irreversibly attached *E. coli* cells have formed small microcolonies (few tens of μm^2). These microcolonies indicated that these *E. coli* cells were in the process of forming a biofilm (Fig. S2). Moreover, it appears that the cells were in contact and covered with some extracellular substances.

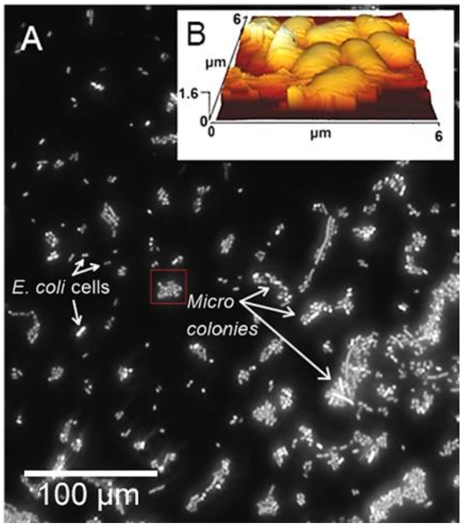

Supplementary Figure 2: *E. coli* cells in the first stages of biofilm formation. (A) Epifluorescent image showing clusters of *E. coli* colonies and corresponding (B) AFM image of one small colony after four hours since washing away any loosely attached cells. Larger biofilms were mot imaged via AFM as the thick EPS matrix was too soft.

Nano-scale images of *E. coli* cells were captured over time without the addition of T4 phages to test the effect of the positively charged LBL on cell morphology. Our images indicate that nano-

scale morphology remained unchanged over time. Hence, we surmise that attaching the cells to the coated LBL layer did not affect the morphology of the cells.

Cell dimensions of uninfected *E. coli* bacteria. Overall, uninfected *E. coli* cells were found to be around 1 µm in diameter and a length that ranged from 2 µm to 3 µm (Fig. S3).

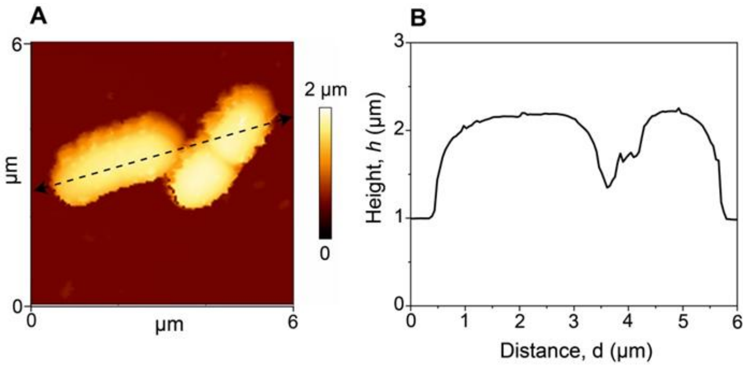

Supplementary Figure 3. Cell dimensions of uninfected E. coli cells (A) AFM topography image of uninfected *E. coli* cells. (B) Height profile (cell cross-section) corresponding to the black dotted line in (A). Similar images were captured for numerous bacteria.

Supplementary Methods

Calculation of the cell elasticity of a single E. coli cell. To calculate the elasticity of a *E. coli* cell, E, the cell was modeled as a cylinder, as depicted in Fig. S4 A.

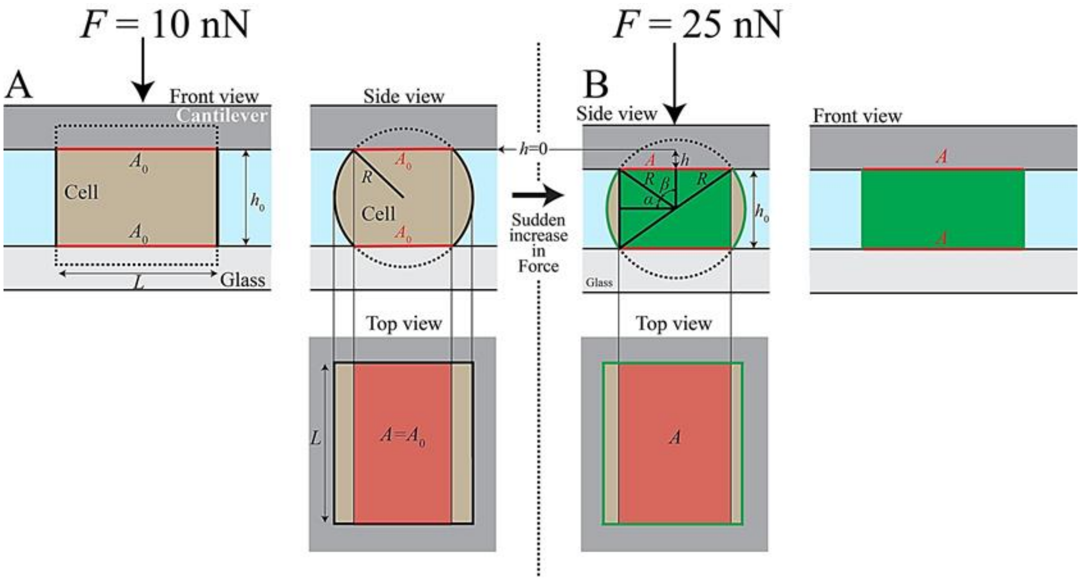

Supplementary Figure 4. **Schematic description of the model used to calculate cell deformation.** Using a tip-less AFM cantilever, a sudden change in force was applied on a separated *E. coli* cell to measure the elasticity, E. (A) The cell was modeled as a cylinder with a base radius R, and length, L. As the cantilever applied force on the cell, as a first order approximation, it was assumed that the parts of the cell that was not in contact with the cantilever, was not deformed; however, the contact area with the cantilever, A (red), increases with F, i.e., the applied pressure on the cell decreased with the cantilever indentation, h.

The strain response of a single cell to a sudden change in force was modeled using Kelvin-Voigt in addition to water permeability through the cell membrane:

$$1-\frac{h}{h_0}=1-\frac{F}{A\cdot E} \qquad \text{(S1)}$$

where h_0 is the height of the cell measured by AFM, h is the indentation of the cantilever into the cell (Fig. S3 B), F is the force that was applied by the AFM cantilever on the cell, and A is the cantilever-cell contact area that increases with h (red in Fig. S4).

Based on the simplified model in Fig. S5, A was estimated using the following equation:

$$A = A_0 + L\sqrt{2Rh - h^2}, \qquad \text{(S2)}$$

where A_0 (estimated parameter) is the cantilever-cell contact area before the sudden change in F (at $t = 0$), the angles α and β are depicted in Fig. S4 B and they are given by $\sin\alpha = \left(\frac{h_0}{2} - h\right)/R$ and $\cos\beta = \frac{R-h}{R}$, R is the radius of the cylinder base, and L is the cell length (Fig. S4 A and B).

Fig. S5 shows typical deformation/strain measurements of non-infected *E. coli* cell and after an hour and a half (~90 min) since the addition of T4 to the *E. coli* cell culture (infected cell). The red circles are the calculated deformations for the two cells at *t*=0 and at *t*=30 s.

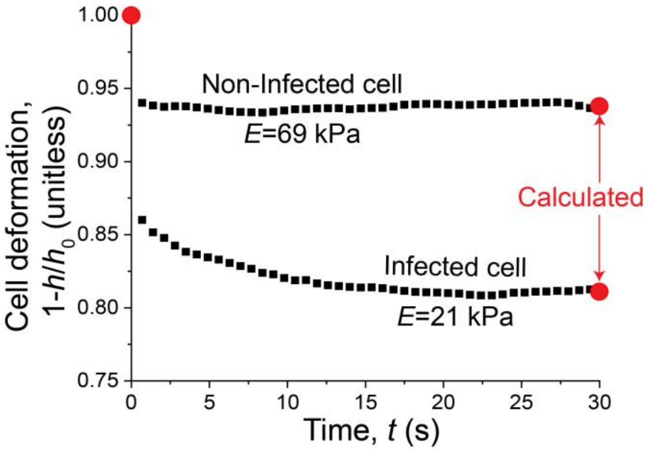

Supplementary Figure 5: Deformation/stain response of *E. coli* cells to a sudden change in stress/force. The black rectangles are the measured data, and the red circles were calculated using Eq. S1 at *t*=0 and at *t*=30 s.

Imaging the physiological state of E. coli cells using following T4 phages addition. The Live/Dead kit (Invitrogen, ThermoFisher Scientific, United States) is commonly used to estimate the physiological state of bacterial cells. Briefly, the kit is based on two molecules with specific

florescence spectra and different penetration efficacy into the cell. Syto9 is a small, fluorescently green (Ex. 485 nm and Em. 498 nm) molecule that penetrates cells irrespective of the state of the cell membranes (REF). Propidium iodide (PI) exhibits red fluorescence (Ex. 535 nm,Em. 617 nm) penetrates the bacterial cell only if the membranes are compromised (REF) . Hence, live cells will appear to be green, while dead cells (or with impair cell-wall) will appear red (Fig. S1). Additional information is provided by the Invitrogen (USA) for L7007 Live/Dead® BacLight Bacterial Viability Kit).

Experimental set up for the attachment of E. coli cells on lipid bilayer (LBL) surface. *E. coli* bacteria must be immobilized to an AFM glass coupon to visualize cells and while conducting mechanical measurements in real time and *in-situ* (namely in phosphate-buffered saline, PBS) using AFM. Since the AFM glass coupon and the surface of the bacterial cells are often negatively charged, it is common to use a support layer which is positively charged to irreversibly attach the cells. However, it is highly important that the coated support layer will not cause physiological stress to the attached cells. Further, reducing the effect of the support layer on the overall elasticity and thickness of attached bacteria is critical to gain equate force measurements. Here, we adopted a novel approach to immobilize bacteria on the surface by employing a net positive surface charge lipid bilayer, LBL (Fig. 1). Bellow we provide a schematic overview of the steps applied before visualizing and analyzing the samples by AFM (Fig. S5). Coating the glass AFM coupon by LBL requires that the surface will be thoroughly cleaned (Fig. S6 A). Thus the glass surface was washed with acetone, ethanol and Milli-Q water, dried with N_2 gas followed by UV treatment for 10 min. The clean AFM coupon was then coated by a positively charged LBL, additional information is provided in the main text (Fig. S6 B). *E. coli* cells (at an exponential phase) were deposited on the surface for 8 hours. The AFM coupon

was then intensively washed to remove all the cells that were loosely attached (Fig. S6 C). AFM coupon with the attached cells was then mounted into the AFM biocell (Fig. S6 D) before the initiation of the experiments.

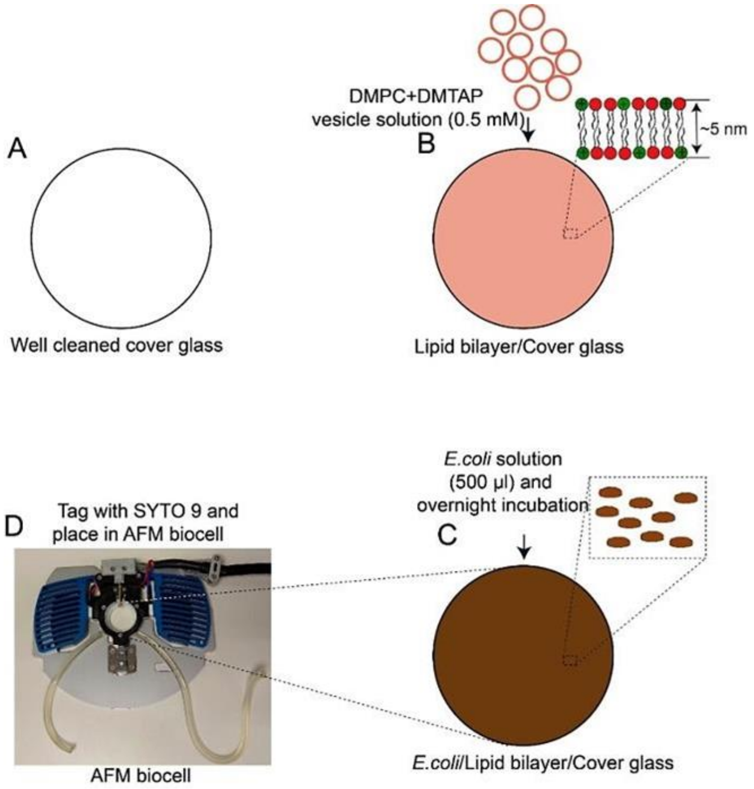

Supplementary Figure 6. Schematic illustration of the experimental setup used to attach *E. coli on* an AFM glass coupon. The main steps included: (A) thorough cleaning of an AFM glass coupon; (B) formation of a positively surface charged LBL on the AFM glass coupon; (C) attachment of *E. coli* bacteria to the LBL substrate; (D) Assemble the coupon with the attached cells into the biocell before visualization and force measurements.
